# Biodegradable and Drug-Eluting Inorganic Composites Based on Mesoporous Zinc Oxide for Urinary Stent Applications

**DOI:** 10.3390/ma13173821

**Published:** 2020-08-29

**Authors:** Marco Laurenti, Marta Grochowicz, Elena Dragoni, Marco Carofiglio, Tania Limongi, Valentina Cauda

**Affiliations:** 1 Department of Applied Science and Technology, Politecnico di Torino, Corso Duca degli Abruzzi 24, 10129 Torino, Italy; marco.laurenti@polito.it (M.L.); or elena.dragoni@gmail.com (E.D.); marco.carofiglio@polito.it (M.C.); tania.limongi@polito.it (T.L.); 2 Department of Polymer Chemistry, Institute of Chemical Sciences, Faculty of Chemistry, Maria Curie Sklodowska University, Gliniana 33, 20-614 Lublin, Poland; mgrochowicz@poczta.umcs.lublin.pl or

**Keywords:** mesoporous zinc oxide, polyHEMA, hydrogel, drug-eluting stent, pH-triggered drug release

## Abstract

Conventional technologies for ureteral stent fabrication suffer from major inconveniences such as the development of encrustations and bacteria biofilm formation. These drawbacks typically lead to the failure of the device, significant patient discomfort and an additional surgery to remove and replace the stent in the worst cases. This work focuses on the preparation of a new nanocomposite material able to show drug elution properties, biodegradation and eventually potential antibacterial activity. Poly(2-hydroxyethyl methacrylate) or the crosslinked poly(2-hydroxyethyl methacrylate)-co-poly(acrylic acid) hydrogels were prepared by the radical polymerization method and combined with a biodegradable and antibacterial filling agent, i.e., flower-like Zinc Oxide (ZnO) micropowders obtained via the hydrothermal route. The physico-chemical analyses revealed the correct incorporation of ZnO within the hydrogel matrix and its highly mesoporous structure and surface area, ideal for drug incorporation. Two different anti-inflammatory drugs (Ibuprofen and Diclofenac) were loaded within each composite and the release profile was monitored up to two weeks in artificial urine (AU) and even at different pH values in AU to simulate pathological conditions. The addition of mesoporous ZnO micropowders to the hydrogel did not negatively affect the drug loading properties of the hydrogel and it was successfully allowed to mitigate undesirable burst-release effects. Furthermore, the sustained release of the drugs over time was observed at neutral pH, with kinetic constants (k) as low as 0.05 h^−1^. By exploiting the pH-tunable swelling properties of the hydrogel, an even more sustained release was achieved in acidic and alkaline conditions especially at short release times, with a further reduction of burst effects (k ≈ 0.01–0.02 h^−1^). The nanocomposite system herein proposed represents a new material formulation for preparing innovative drug eluting stents with intrinsic antibacterial properties.

## 1. Introduction

Nowadays, the use of ureteral stents is a conventional and routine practice. However, it shows a huge number of drawbacks due to the absence of material optimization and lack of advanced performances, especially in comparison with the advancement obtained in other fields such as cardiovascular stents.

The main limitations of current ureteral stents are (i) the encrustation of the system induced by the precipitation of inorganic salts from urine and (ii) the formation of bacteria biofilm, with consequent antibiotic resistance, persistence of the infections, morbidity, urinary retention, ureteral damage and in the worst cases pyelonephritis and sepsis [1,2]. All these drawbacks lead to significant patient discomfort (pain, urgency, frequency) and a second surgery to remove or replace the stent, even often supported by an alongside antibiotic, analgesic or other drug treatments [3]. As a result of frequent re-hospitalization, an increase in public health costs are also evidenced [4].

In the last decades, several approaches have been investigated to modify the stent itself including a new device design, optimization of the polymeric formulation, the deposition of surface coatings to prevent bacteria and inorganic encrustation and other advanced applications such as drug-elution or complete stent biodegradation to avoid further re-hospitalization [5]. In particular, the use of functional coatings has been widely considered and various materials turned out to be a valid solution for preventing both the adhesion of bacteria and the formation of inorganic encrustation to the stent surface. These included the use of antimicrobial silver [6], hydrogels (like Hydromer^®^) [7], heparin [8] and diamond-like amorphous carbon coatings [9] to name only a few. On the other side, biodegradable and drug eluting stents made by polylactic acid (PLA) polymers have been tested in animal models with appreciable results [10]. However, the considered stent only partially degraded and small polymer fragments remained in the ureter [11], inducing the obstruction to the urine flow.

Recently, the use of nanostructured multifunctional materials for biomedical applications attracted great attention as these can represent an alternative approach with respect to most of the conventional ones investigated right now [12,13,14]. Among nanomaterials, Zinc Oxide (ZnO) is one of the most studied [15,16]. ZnO can be used for many applications thanks to its very interesting physical and chemical properties. Moreover, it can be easily prepared in several high-surface-area morphologies by low-cost hydrothermal methods [17,18]. Some of them include nanotubes [19] and nanowires [20,21], nanobelts [22], nanorings [23], flower-like particles [24,25], nanosprings [26], multipods [27,28] and nanoparticles [29]. ZnO is a “generally recognized as safe” (GRAS), white, odorless inorganic solid compound. ZnO also exhibits intrinsic antibacterial properties [30], thereby paving the way to its possible use as intrinsically-antimicrobial material. The antibacterial activity of ZnO is mainly ascribed to the following mechanisms [31]: reactive oxygen species (ROS) generation, release of Zn^2+^ cations, and electrostatic interactions between ZnO nanomaterials and the bacterial surface. Despite the potential role in determining ZnO antibacterial efficacy, the release of Zn^2+^ ions can also induce undesirable cytotoxicity against healthy cells and their release must be properly optimized and monitored to guarantee biocompatibility at the same time [29,32]. Especially for biomedical implants or drug delivery devices, the control over ZnO particle size, morphology, interparticle porosity and surface chemistry is crucial to achieve antibacterial properties and biocompatibility towards healthy cells at the same time [30]. Another important aspect of ZnO is its biodegradability, as it is easily soluble in water solution at pH below 5.5 or in water solution rich in salts, such as phosphates [33]. It is, therefore, clear that the use of ZnO-based materials in the field of ureteral stents is not only novel but can also give many advantages.

To overcome some of the abovementioned limitations, ZnO has also been incorporated into various polymer materials such as Polyvinyl alcohol (PVA), Poly(methyl methacrylate) (PMMA), Polypropylene (PP), Polystyrene (PS), Polydimethylsiloxane (PDMS) or photocurable resins [28,33,34,35]. The advantages of this approach are manifold, especially for biomedical applications. For example, the release of potentially harmful Zn^2+^ cations can be properly limited without vanishing the corresponding antimicrobial action. Moreover, a sustained release of drugs over time and a limitation of undesirable burst-release effects typically observed for pure ZnO-based systems [36] could be achieved. Finally, the addition of ZnO to the polymer matrix can result in a lower friction coefficient with respect to the pristine polymer, useful to overcome the problem of discomfort of traditional ureteral stents [37].

This work aims to propose a new composite material which combines mesoporous ZnO micropowders with a hydrogel matrix in view of fabricating smart and innovative drug-eluting ureteral stents. The composite material is designed to obtain efficient anti-inflammatory drug release, biodegradation of the device itself due to zinc cations release and thus also intrinsic antibacterial properties [30,33]. To achieve such a challenging goal, poly(2-hydroxyethyl methacrylate) (polyHEMA) or the crosslinked co-polymer poly(2-hydroxyethyl methacrylate)-co-poly(acrylic acid) (poly(HEMA-co-AA)) hydrogels were selected in view of their biodegradability and swelling properties. Mesoporous ZnO micropowders, prepared by low-cost hydrothermal route, were selected as filling agent in view of their intrinsic antimicrobial and biodegradation properties coupled with a high surface area and high level of porosity in the size range of mesopores (as defined by the International Union of Pure and Applied Chemistry, IUPAC [38]), ideal for drug uptake and retention. The composites were prepared by incorporating different percentages of the mesoporous ZnO powders into the polymeric composition. Then, two different anti-inflammatory drugs (Ibuprofen and Diclofenac, both in salts form) were loaded and the corresponding release profiles were investigated in artificial urine, even at different pH to simulate pathological conditions.

## 2. Materials and Methods

### 2.1. Materials Preparation

#### 2.1.1. Synthesis of Flower-Like ZnO Mesoporous Microparticles

Mesoporous ZnO microparticles have been prepared using a low-cost, hydrothermal sol-gel synthesis approach. All the chemical reagents have been used as purchased and without any further purification. In total, 5.58 g of potassium hydroxide (KOH, Sigma Aldrich, Darmstadt, Germany) and 14.8 g of zinc nitrate hexahydrate (Zn(NO_3_)_2_·6H_2_O, Sigma Aldrich) were both dissolved separately in 100 mL of bidistilled water under vigorous magnetic stirring conditions. Then, the zinc nitrate solution was added dropwise to the KOH solution under vigorous magnetic stirring to allow the reaction between precursors and the formation of a sol. After gelation, the gel was placed in a closed Teflon bottle and treated at 70 °C for 4 h. After this time, a ZnO powder precipitated at the bottom of the vessel. By filtration, it was carefully separated from the solution (pH 14) and washed several times with demineralized water until pH neutralization. Finally, the ZnO powders were air dried at 60 °C overnight. At the end of the preparation, 3.0 g of ZnO powders can be produced.

#### 2.1.2. Synthesis of Poly(2-hydroxyethyl methacrylate) and Poly(HEMA-co-AA)

**Scheme 1:**
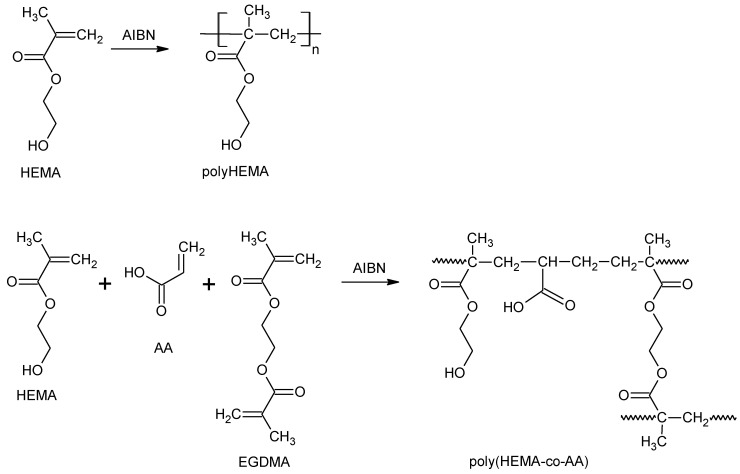
The reaction scheme for the crosslinked polymers/hydrogel formation.

In total, 3.0 g of poly(2-hydroxyethyl methacrylate, pHEMA) hydrogel have been prepared by radical polymerization method. In total, 5.0 g of 2-Hydroxyethyl Methacrylate (HEMA) and 0.1 g of initiator 2,2-Methylpropionitrile (AIBN, 98%) were mixed together in 50 mL of toluene as solvent. To avoid the inhibition of polymerization due to oxygen, the solution was first placed under continuous nitrogen flowing. Then, radical polymerization was started by activating the initiator at 70 °C under magnetic stirring for 6 h. At the end of the reaction (Scheme 1), polyHEMA is formed and toluene is removed by low-temperature distillation at 70 °C under reduced pressure conditions (15·10^−3^ bar). Poly(HEMA-co-AA) copolymer has been prepared by combining HEMA monomer solution with 5% vol. of acrylic acid (AA) monomer and ethylene glycol dimethacrylate (EGDMA) used as a crosslinking agent (Scheme 1). Further details about the preparation of the polymers can be also found in ref. [39].

#### 2.1.3. Synthesis of Composites Based on polyHEMA@ZnO and poly(HEMA-co-AA)@ZnO

The polymer@ZnO composites have been prepared by mixing the monomer solution (HEMA or HEMA-co-AA) with mesoporous ZnO micropowders in different amounts (4 mg and 40 mg), corresponding to 0.1 wt.% and 1 wt.% ZnO/polymer composite samples. In order to improve the ZnO dispersion, pre-polymerization of the solution with monomer and mesoporous ZnO powders is carried out by heating the solution in a glass tube on a hot plate at 90 °C for 30 min and until it became viscous. In total, 1% of Benzoyl Peroxide was used as an initiator (percentage relates to the total amount of monomers). The reaction is immediately interrupted by putting the beaker in ice. A rectangular silicon rubber (PDMS, polydimethylsiloxane) mold (3 cm × 2 cm × 0.3 cm in dimensions) is filled with 4 mL of the pre-polymerized solution, clamped between two Teflon plates and heated in oven for 4h at 70 °C to complete polymerization. In the specific, polyHEMA@ZnO composites have been prepared starting from HEMA monomer mixed with 1% vol. of initiator and ZnO powders. Poly(HEMA-co-AA)@ZnO were obtained from HEMA monomer, 1% vol. of AA and mesoporous ZnO powders. In the latter case, ethylene glycol dimethacrylate crosslinker (5% vol.) was also added to avoid degradation of the copolymer and improve the stability of the system. A picture representative of polyHEMA@ZnO composite sample is reported in Figure S1 of the Supporting Information.

The maximum and minimum ZnO weight percentages used in this work have been selected according to ref. [40], in order to provide the correct balance between antibacterial behavior and safety for healthy human cells of the ureter’s epithelium. This will be motivated by further zinc cations release tests and the consideration reported in the Results and Discussion section.

Zinc cation release tests were performed by soaking pristine ZnO and polyHEMA@ZnO composite samples in 700 μL of Dulbecco’s Modified Eagle Medium (DMEM) completed with 10% *v/v* of fetal bovine serum. The samples were prepared according to ref. [41] and incubated at 37 °C in orbital shaking conditions for different times (i.e., 2 h, 8 h, 3 days and 7 days). The free zinc cations concentration in the supernatant was evaluated through atomic absorption spectroscopy.

### 2.2. Ibuprofen and Diclofenac Uptake and Release

Diclofenac (DF) and Ibuprofen (IB) were selected as nonsteroidal anti-inflammatory drugs and the loading into the considered samples has been performed as follows. Both the drugs have been purchased from Sigma in sodium salt form and dissolved in water (final concentration 10 mg/mL) under vigorous magnetic stirring. The samples have been soaked into the drug solution at 37 °C to support drug solubility and under stirring conditions to prevent the formation of concentration gradients. A gas-tight cap was put on the vessel during the overall loading experiments to avoid evaporation. After 4 h, the samples have been removed and dried in an oven at 50 °C. The amount of loaded drug (11.6 ± 1.8 mg for DF and 15.3 ± 3.0 mg for IB) was estimated by weighing each dried sample before and at the end of drug uptake experiment.

The release experiments have been carried out by soaking each sample in artificial urine solution (50 mL) at physiological conditions (pH 7.4) but also at alkaline (pH 9) and acid (pH 4.5) ones to simulate pathological conditions. Artificial urine has been prepared according to ref. [42], as following: 500 mL of bidistilled water were heated at 37 °C and maintained under continuous magnetic stirring. Then, all the reagents (KCl 0.2 g/L, NaCl 8.0 g/L, Na_2_HPO_4_ 1.14 g/L, KH_2_PO_4_ 0.2 g/L) were added. After their complete dissolution, HCl was added until neutral pH was achieved. Then, the solution obtained was transferred in 1 L-flask and extra bidistilled water was added until reaching 1 L of artificial urine. At the end, the whole volume was transferred inside a 2L-bottle and stored in the fridge at +4 °C. When required, alkaline and acid pH conditions were obtained by adding drop-by-drop sodium hydroxide (NaOH, 1 M) and hydrogen chloride (HCl, 1 M), respectively, until reaching the desired pH value.

The samples have been soaked in orbital shaking conditions at 200 rpm (Incubating orbital shaker, Professional 3500, VWR International Ltd., Radnor, PA, USA) and maintained at a physiological temperature of 37 °C. At specific points of time (1 h, 2 h, 3 h, 4 h, 5 h, 6 h, 24 h, 48 h, 7 days, 14 days), 100 µL of the release solution was withdrawn and analyzed with ultraviolet-visible (UV-vis) spectroscopy to estimate the amount of released drug. All of the uptake/release experiments have been performed in triplicate.

### 2.3. Characterization Techniques

Field-emission Scanning Electron Microscope (FESEM) and Energy-dispersive X-ray (EDX) spectroscopy were carried out by using an FESEM MERLIN from ZEISS (Oberkochen, Germany). The crystalline structure was studied by X-Ray Diffraction (XRD), with a Panalytical X’Pert PRO diffractometer (Malvern Panalytical S.r.l., Milan, Italy). Cu Kα monochromatic radiation was used as the X-ray source (λ = 1.54059 Å, 40 kV, 30 mA). The diffraction patterns were collected in the 5°–80° range with a step size of 0.02° and the acquisition time was set to 100 s per step.

Nitrogen sorption isotherm (Quadrasorb SI, Quantachrome, Anton Paar QuantaTec Inc., Boynton Beach, Florida, USA) was applied to measure the porosity of the mesoporous flower-like ZnO micropowder. The Brunauer–Emmett−Teller (BET) specific surface area was applied from by multipoint method within the relative pressure range of 0.1−0.3 p/p_0_. The density functional theory (DFT) model was also applied to estimate the pore size distribution of the flower-like ZnO particles.

UV-VIS absorbance spectra of drug release solutions (100 µL) were collected in the range 200–800 nm, by means of a 96-well quartz plate and a microplate reader (Multiskan™ FC Microplate Photometer, from ThermoFisher Scientific, Waltham, MA, USA). All of the UV spectra were background subtracted. Fourier Transformed infrared (FTIR) analyses were performed in attenuated total reflectance (ATR) mode with a Nicolet 5700 FTIR Spectrometer (ThermoFisher, Waltham, MA, USA). ATR-FTIR spectra acquisition was performed with 4 cm^−1^ resolution and 16 scans accumulation. Thermal Gravimetric Analysis (TGA) was performed with STA 449 Jupiter F1 (Netzsch, Selb, Germany). TGA measurements with the mass of ≈10 mg were carried out in the temperature range from 30 to 600 °C at the heating rate of 10 K/min under air atmosphere (gas flow 20 mL/min). The empty Al_2_O_3_ crucible was used as a reference.

## 3. Results and Discussion

### 3.1. Characterization of Flower-Like ZnO Microparticles

**Figure 1:**
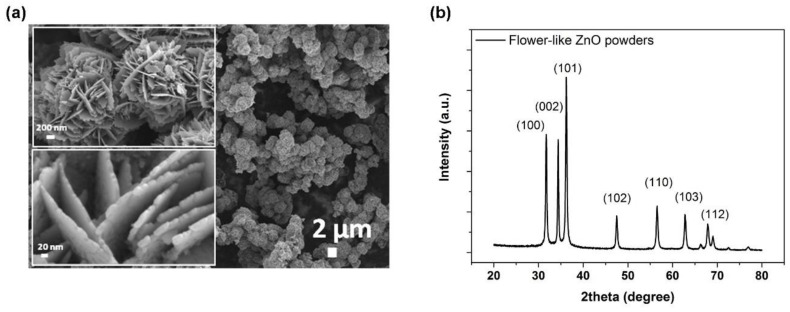
(**a**) FESEM images and (**b**) XRD pattern of flower-like ZnO microparticles obtained by hydrothermal method.

The morphology and the crystalline structure of the mesoporous flower-like ZnO microparticles were analyzed by means of FESEM, nitrogen sorption measurements and XRD. Figure 1a clearly highlights that the as-prepared ZnO powders consisting of spherical particles (average diameter 2.5 μm) with a flower-like morphology, due to a lateral growth of nanometer-sized crystalline facets (average thickness 15 nm). A relatively high surface area (19.6 m^2^g^−1^) and pore diameter with average size of 4 nm are evidenced from the nitrogen sorption isotherm (see Figure S2 of the Supplementary Materials.) and confirmed by the previous literature [25,43]. These pores, as well as the flower-like morphology, are responsible for the relatively high surface area and also act as preferential adsorption sites of the used drugs.

The XRD pattern shown in Figure 1b evidences the polycrystalline structure of the as-prepared mesoporous ZnO micropowders which crystallize in the hexagonal wurtzite structure. The diffraction pattern presents the reflection peaks typical of this crystalline phase and can be indexed according to JCPDS (file No. 80-0074) as (100) at 31.82°, (002) at 34.54°, (101) at 36.42°, (102) at 47.46°, (110) at 56.74°, (103) at 62.6° and (112) at 67.8°. The Debye–Scherrer model applied to the (101) diffraction peak leads to an average crystal size of 15 nm, which fairly corresponds to the thickness of the flower-like petals, demonstrating their nanometric nature.

The growth of this peculiar flower-like morphology is inferred to the chemical reaction used for the preparation of the ZnO powders [37]. During the hydrothermal synthesis, the coordination/electrostatic interactions allow hydroxyl groups (OH^−^) to bind to Zn^2+^ metal cations. The reaction between Zn^2+^ ions and OH^−^ groups form the growth unit $\mathrm{Zn}{\left(\mathrm{OH}\right)}_{4}^{2-}$, from which ZnO can be obtained by dehydration, is as follows [28]:

Zn^2^^+^ + 2OH^−^ → Zn(OH)_2_ (1)

(2) $${\mathrm{Zn}(\mathrm{OH})}_{2}\;+\;2{\mathrm{OH}}^{-}\;\to \;\mathrm{Zn}{\left(\mathrm{OH}\right)}_{4}^{2-}$$

(3) $$\mathrm{Zn}{\left(\mathrm{OH}\right)}_{4}^{2-}\;\to \;2\mathrm{ZnO}\;+\;2H_{2}O\;+\;4{\mathrm{OH}}^{-}$$

Hence, the formation of $\mathrm{Zn}{\left(\mathrm{OH}\right)}_{4}^{2-}$ units and the concentration of OH^−^ in the reaction are crucial in determining the growth rate of different crystal faces and the final ZnO morphology.

### 3.2. Characterization of ZnO/2-Hydroxyethyl Methacrylate-Based Composite Systems

**Figure 2:**
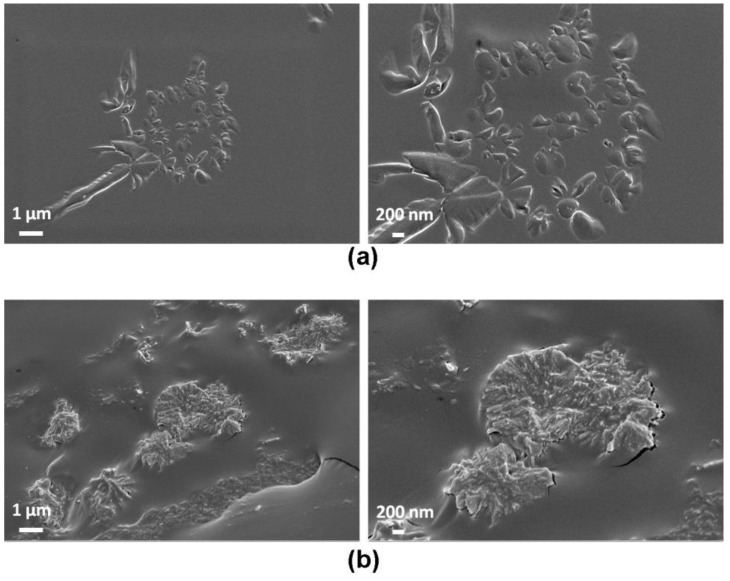
Morphological analysis of polyHEMA@ZnO composite samples incorporating different ZnO amounts: (**a**) ZnO_0.1 wt.%; (**b**) ZnO_1 wt.%.

The incorporation of flower-like ZnO microparticles within the composite material was first assessed from the morphological standpoint. Figure 2a shows the FESEM image corresponding to the polyHEMA@ZnO composite sample incorporating the lowest amount of ZnO (0.1 wt.%) used in this work. In this case, only some traces of the ZnO powders imprinted in the polymer matrix were identified and it was due to the use of a reduced quantity of ZnO. Instead, the flower-like particles were physically localized in the case of the composite containing a higher quantity of ZnO powders (1 wt.%). Figure 2b reports the corresponding FESEM image and clearly shows individual flower-like ZnO microparticles correctly incorporated inside the polymer. Similar results were obtained in the case of the copolymer (polyHEMA-co-AA), as shown in Figure S3 of the Supplementary Materials.

The presence of ZnO powders was further confirmed by chemical composition analyses. The detection of Zn traces was confirmed by EDX analyses (Figure S4 of Supplementary Materials), with sample polyHEMA@ZnO_1% showing the higher Zn amount (0.03 at. %). The detection of the Zn element was possible on sample polyHEMA@ZnO_0.1% as well, even though the detected amount was even smaller (0.01 at.%). Even if the presence of ZnO has been only slightly detected by EDX, it is still possible to notice that polyHEMA@ZnO_1% shows a higher atomic percentage of Zn compared to polyHEMA@ZnO_0.1%. This result is thus in line with the formulation of the composites.

**Figure 3:**
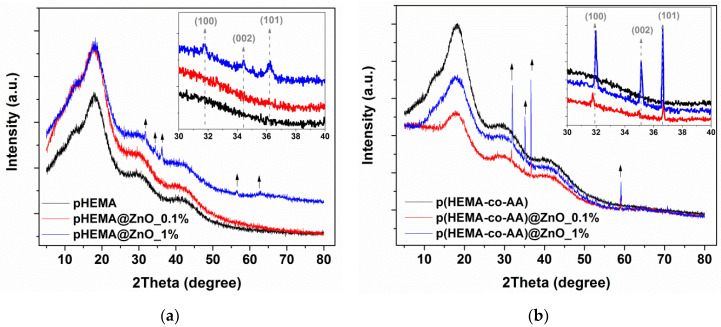
XRD patterns of (**a**) polyHEMA@ZnO and (**b**) poly(HEMA-co-AA)@ZnO composite samples incorporating different amounts of ZnO. For each panel, arrows indicate the diffraction peaks coming from wurtzite ZnO phase. Insets show a magnification of the XRD region showing the rise of the main peaks due to (100), (002) and (101) ZnO phase.

The actual incorporation of the ZnO powders for all the samples’ typologies was further corroborated by XRD measurements, which are shown in Figure 3. The detection of the main diffraction peaks due to ZnO was observed in all the cases, independent of the polymer matrix (polyHEMA or the corresponding copolymer). Insets of each panel in Figure 3 further underline that ZnO has been correctly incorporated, as witnessed by the presence of the strong diffraction contributions coming from (100), (002) and (101) peaks due to the wurtzite ZnO phase, and positioned at 31.82°, 34.54° and 36.42°, respectively. It can be also seen that with increasing the amount of ZnO powder, the intensity of the above-mentioned peaks increases consistently, as expected. Actually, in the XRD pattern of the samples incorporating the lowest amount of ZnO (0.1 wt.%, red lines) the peaks due to ZnO are slightly noticeable. On the other hand, in the sample with 1 wt.% of ZnO (blue lines), the peaks are more clearly visible.

**Figure 4:**
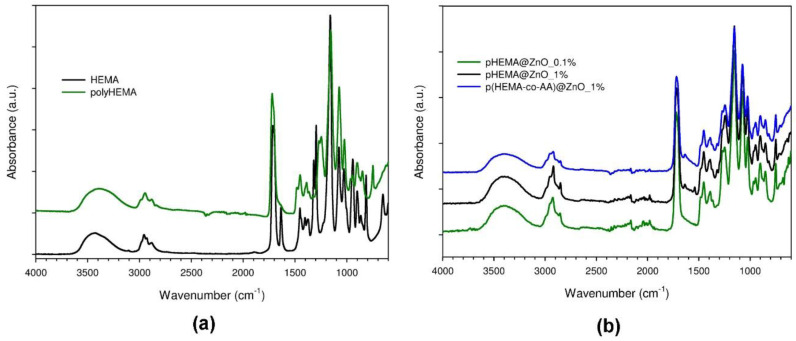
(**a**) FTIR spectra of HEMA monomer and of polyHEMA hydrogel obtained after radical polymerization; (**b**) Comparison of FTIR spectra of composite samples incorporating different percentages of ZnO.

The chemical structure of the investigated composite samples was assessed by means of FTIR spectroscopy. Figure 4a shows the FTIR spectra of the monomer used for the polymerization and of the polyHEMA hydrogel matrix, while Figure 4b shows the spectra of the corresponding composite samples including ZnO in different amounts. The only difference between the monomer and polyHEMA is the presence of H₂C=C groups in the first one and represented by two peaks at around 1600 cm^−1^ and 1000 cm^−1^. These are more intense in the spectrum of the monomer while are slightly pronounced in the second case. This aspect allows to state that the polymerization of the starting monomer occurred correctly. However, it can be also noticed that a small amount of the monomer did not react during the polymerization, since the vibration peaks of H₂C=C groups did not completely disappear from the spectrum of polyHEMA, especially the one positioned at around 1600 cm^−1^. Other peaks are detected as well, both in the monomer and in the polyHEMA sample. The peak over 3500 cm^−1^ relates to OH groups. The peak over 3000 cm^−1^ is associated with CH_3_ and CH_2_ groups. In the low wavenumber region, the peak over around 1700 cm^−1^ is associated to C=O groups while the peak over around 1250 cm^−1^ corresponds to C=C with CH_2_ groups.

Concerning the FTIR spectra of the composite samples (Figure 4b), the creation of hydrogen bonding with another compound (in our case ZnO) tends to decrease the frequency of C=O stretching. The formation of carboxylic acid salts due to the surface interaction between pHEMA and ZnO is witnessed by the characteristic band at 1650 cm^−1^ due to CO_2_^−^ asymmetric stretching vibration, which also gives rise to a band in the range 1440–1335 cm^−1^ with two characteristic peaks. Together with this C-O stretching, the O-H deformation vibration can be found in the same region, both due to the carboxylic acid of pHEMA and hydroxyl groups from the ZnO surface [44]. The presence of a broad band in the range 950–850 cm^−1^ due to Zn-OH mode is observed in the FTIR spectrum of the ZnO powders (Figure S5 of Supplementary Materials) and also in the spectra of the composite samples.

**Figure 5:**
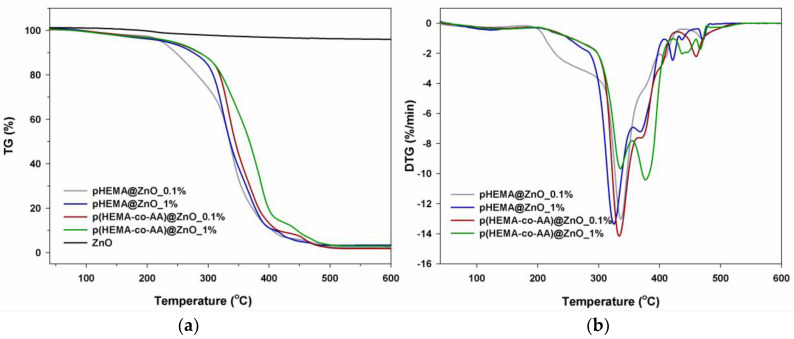
Thermal degradation curves of polymer-ZnO composites as well as ZnO powders as control sample (curve in black). (**a**) Thermogravimetry (TG) analysis and (**b**) Differential thermogravimetric (DTG) measurement.

TGA (Figure 5) was performed for all the samples’ typologies to evaluate the thermal degradation behavior of the considered materials. Actually, an appropriate knowledge of the thermal resistance of polymer-based composites is important because of the processing conditions used. From the differential thermogravimetric (DTG) curves (Figure 5) it can be seen that the sample degradation occurs via a multistep process, as evidenced by the corresponding distinct peaks. The first step from room temperatures to around 160 °C is due to the evaporation of non-bonded water, other volatile compounds and residual unreacted monomers still present in the composites, as discussed before. The second step takes place at around 250 °C and is the most pronounced for polyHEMA with the smallest amount of ZnO. The decomposition of polyHEMA network as well as the copolymer networks start from around 200 °C. The higher thermal resistance (expressed as 2% of mass loss) for copolymer composites, with respect to the polymeric ones, is due to their crosslinked structure. The next two steps with maximum at about 330 °C and 370 °C are due to the main decomposition of polymer networks in the whole mass. The previous studies showed that depolymerization to the monomer is the dominant decomposition process for pHEMA, especially at lower temperatures [45,46]. However, the presence of ZnO powder in the polymeric samples shifts their thermal resistance towards higher temperatures.

**Figure 6:**
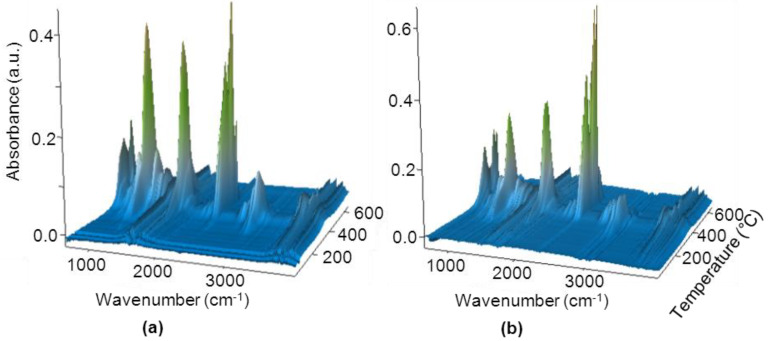
The 3D FTIR spectra for (**a**) polyHEMA@ZnO_0.1%; (**b**) crosslinked poly(HEMA-co-AA)@ZnO_0.1%.

**Figure 7:**
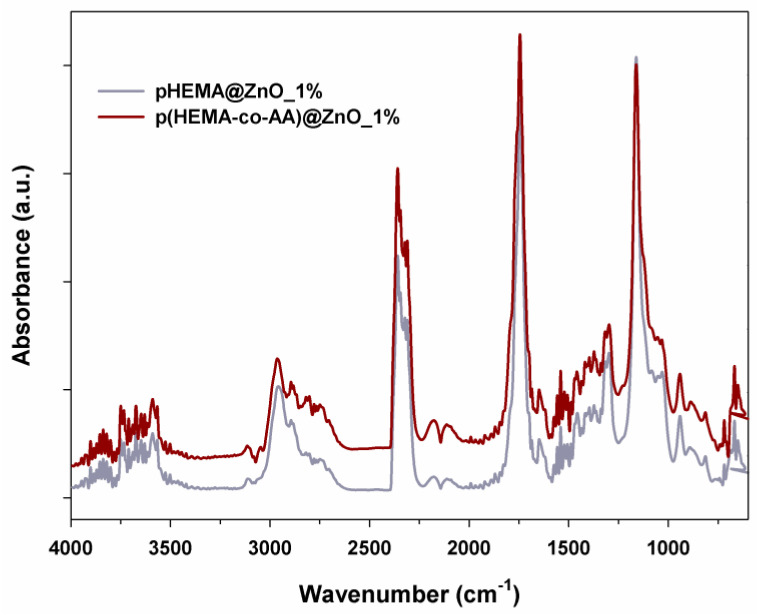
The FTIR spectra of the evolved gases from polyHEMA@ZnO_0.1% at 348 °C (grey) and from poly(HEMA-co-AA)@ZnO_0.1% at 348 °C (dark red).

To evaluate the degradation products, the emissions from TGA were evaluated and identified by FTIR analysis. Figure 6 gives a 3D spectrum of gases produced during the thermal degradation, while Figure 7 shows the FTIR spectra obtained from the 3D FTIR ones and evaluated at the maximum degradation temperature.

From these results it can be stated that the degradation of polyHEMA@ZnO and poly(HEMA-co-AA)@ZnO starts from the depolymerization process to HEMA, and is followed immediately by oxidation reactions. The emission of HEMA, water and carbon monoxide and dioxide at about 350 °C, confirms such degradation patterns. However, the intensity ratio of the carbonyl band (at 1745 cm^−1^) to carbon dioxide band (2357 cm^−1^) indicates that the oxidation is dominant process for copolymer samples. At temperatures higher than 370 °C mainly the emission of carbon dioxide and water is observed for all samples what indicates the oxidation processes. In the spectra of Figure 7, the characteristic absorption bands for HEMA at 1745, 1638 and 1162 cm^−1^ are visible. Instead, the bands at 2357, 2311 and 670 cm^−1^ belong to carbon dioxide, while the shoulder on the absorption band at 1745 cm^−1^ is associated to another carbonyl species. Finally, the bands in the regions 3800–3600 cm^−1^ and 1600–1400 cm^−1^ are characteristic of water evaporation.

As stated in the Materials and Methods Section 2.1.3, our intention when compounding the composite materials was to provide the correct balance between antibacterial behavior and safety for healthy human cells of the ureter’s epithelium. In particular, the amount of ZnO used in the composites varies between 0.1 and 1%wt/v.

ZnO at the bulk or micro-sized scale is classified as a GRAS—generally recognized as safe—substance by the US Food and Drug Administration (FDA) [47], but is also one of the most promising inorganic antimicrobial materials [48,49]. However, it can cause potential toxicity to eukaryotic cells in a dose dependent way [40,50,51,52]. Thus, the use of ZnO as an antimicrobial compound requires a careful balance between the material amount and the preservation of healthy living tissues and bacterial species to be killed. In a recent study performed by some of us [30], nanometer-sized ZnO were tested as nanoantibiotics against both Gram-positive and negative bacteria, such as *Escherichia coli* and *Staphylococcus aureus*, respectively. In that study, ZnO nanocrystals have shown at the highest concentration experimented, i.e., 100 µg/mL, great biocompatibility towards healthy cells (in that case pre-osteoblast cells) in terms of differentiation and proliferation of cells, and very promising antimicrobial activity against both *E. coli* and *S. aureus*.

In the present paper, a similar amount of ZnO was used in the composite formulation and allowed us to speculate that we are in the good direction to propose an efficacious biomedical device.

Additionally, a preliminary study about the dissolution behavior of ZnO microparticles in cell culture medium (to mimic the potential toxic environment against eukaryotic cells), even alone or in combination with polyHEMA, was carried out through zinc cation release tests [41], whose results are reported in Figure S6 of the Supplementary Materials. For pure ZnO microparticles, the zinc cations’ concentration slowly increased with incubation time in cell culture medium. The maximum value of concentration was reached after 1 week (90.4 µg/mL), i.e., the maximum incubation time considered in this study. However, this level of released zinc cations may be cytotoxic for human cells [53]. Nevertheless, an interesting aspect arises when the ZnO microparticles are combined together with polyHEMA: a reduction of the zinc cations release was successfully obtained. Specifically, the amount of zinc cations released after incubating the samples for 3 and 7 days is reduced by 74.6% and 80.4% in comparison to that observed for the pure ZnO microparticles.

Despite using a different medium (cell culture medium instead of artificial urine solution) and a low amount of polyHEMA, it can be speculated that, in the case of the composite material discussed in this work, the use of a large amount of polyHEMA in which ZnO particles are dispersed would prevent even more the dissolution of the oxide particles, leading to the cytocompatibility of the system toward epithelial cells. An efficient zinc cation release will both allow the time-dependent biodegradation of the biomedical device and can be responsible of an interesting antimicrobial activity of the proposed composites. Further tests in this direction have to be certainly carried on in a future publication.

### 3.3. Drug Release in Physiological Artificial Urine

**Figure 8:**
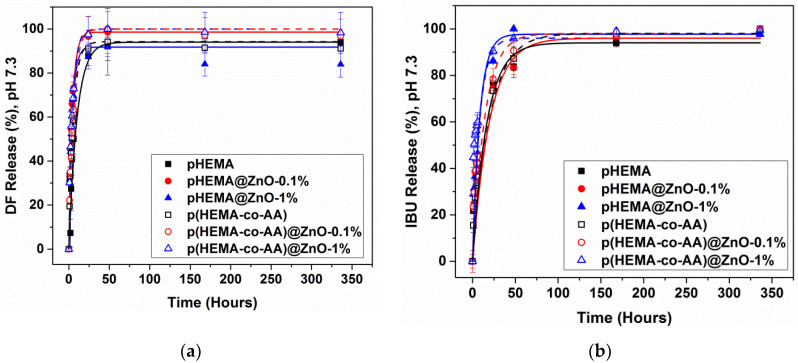
Kinetic of drug release during time, in physiological artificial urine solution: (**a**) diclofenac and (**b**) ibuprofen, both in sodium salts form. Experimental points are reported along with their error bars. All of the experimental points follow a pseudofirst-order kinetic law. Fitting curves are represented by plain lines for pHEMA-based samples and by dashed lines for p(HEMA-co-AA)-based samples.

Figure 8 show the kinetic release profiles obtained for diclofenac (DF) and ibuprofen (IBU) drugs in artificial urine solution at physiological conditions (pH 7.3). All of the release profiles follow a pseudo-first-order kinetic law and the corresponding kinetic parameters (k, kinetic constant; A_48h_, % of drug released after 48 h; A, % of drug released at the end of the experiment) are shown in Table 1. Independently of the considered material, the release kinetic was different between IBU and DF. Figure 8a highlights that DF was completely delivered after 48 h in most of the cases. A different behavior was observed only for pure polyHEMA and poly(HEMA-co-AA) samples, which showed low kinetic constants and a limited burst delivery at the same time. In particular, polyHEMA showed the lowest kinetic constant (k, 0.110 h^−1^) and the maximum DF release (A, 96%) was reached only after 7 days (168 h). It can be also observed that the release kinetic increases according to the amount of ZnO incorporated within the polymer. Actually, both the polymers loaded with 1% ZnO are characterized by the highest kinetic constant.

On the other side, Figure 8b shows a more sustained delivery IBU over time. The release increases continuously, and it is completed after 7 days (168 h) in most of cases; only sample polyHEMA@ZnO_1% reaches the maximum release after 48 h. Concerning the release kinetic, the behavior of the investigated materials is similar to the one observed for DF: the pure polymer materials show a limited burst effect and low kinetic constants, while those incorporating the highest amount of ZnO powders show the fastest IBU release with the composite poly(HEMA-co-AA)@ZnO_1% one, having the highest release kinetic (0.120 h^−1^).

### 3.4. Drug Release in Acid and Alkaline Artificial Urine

**Figure 9:**
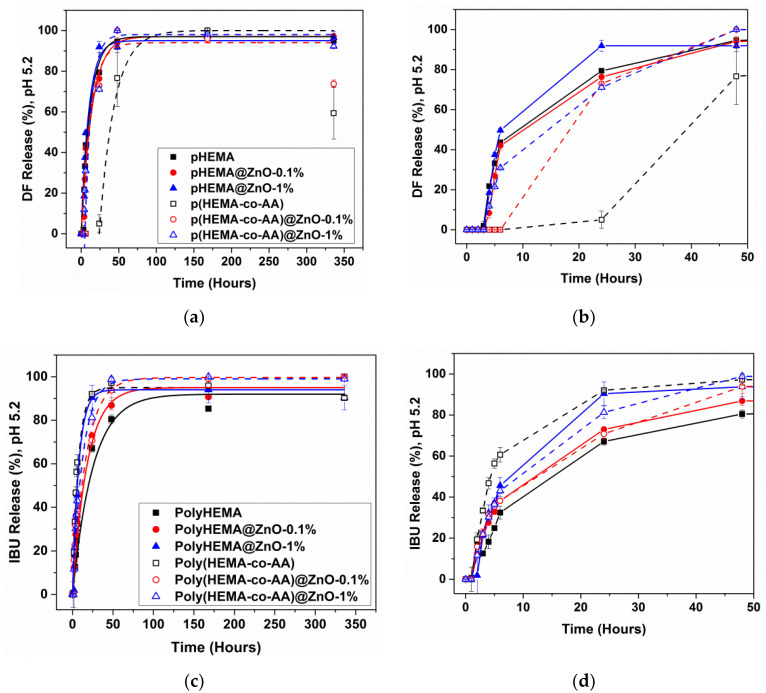
Drug release profiles during time in acidic artificial urine solution: (**a**,**b**) diclofenac and (**c**,**d**) ibuprofen, both in sodium salts form. Experimental points are reported along with their error bars. All of the experimental points follow a pseudofirst-order kinetic law. Fitting curves are represented by plain lines for pHEMA-based samples and by dashed lines for p(HEMA-co-AA)-based samples.

In physiological conditions, the pH value for urine is between 4.5 and 7.5, with a tendency to keep a pH value slightly acid. Figure 9a–d show the kinetic release for both DF and IBU drugs in an acidic artificial urine environment (pH 5.2). In this case, a more sustained release was observed with respect to neutral pH conditions, especially within 48 h, and it is well represented by the kinetic parameters reported in Table 2. If DF is considered, the release is strongly delayed. In particular, when a poly(HEMA-co-AA) sample is considered, DF delivery occurred only after 24 h (Figure 9b), while after 4 h for the other poly(HEMA-co-AA)@ZnO samples. In the case of polyHEMA and polyHEMA@ZnO composites, DF release started at lower release times (after 2 h). The maximum amount of drug released at the end of the experiment was generally lower than 100% and the amount of drug released after 48 h was highly lower with respect to what observed in neutral pH conditions, as discussed before. Similar aspects were found for IBU release, independently of the considered sample. The release of this drug generally started only after 2 h, and the maximum amount of drug released was lower than 100%.

**Figure 10:**
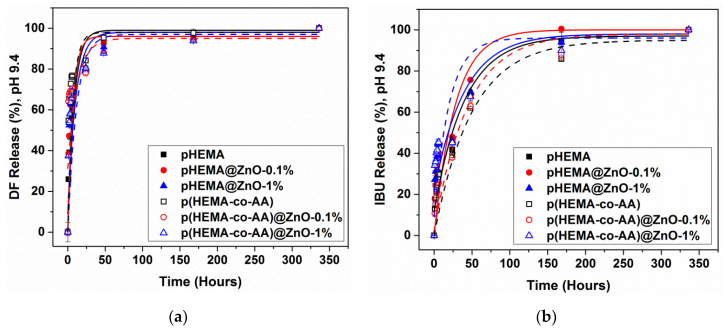
Kinetic of drug release during time, in alkaline artificial urine solution: (**a**) diclofenac and (**b**) ibuprofen, both in sodium salts form. Experimental points are reported along with their error bars. All of the experimental points follow a pseudo first-order kinetic law. Fitting curves are represented by plain lines for polyHEMA-based samples and by dashed lines for poly(HEMA-co-AA)-based samples.

To simulate pathological conditions, the pH of artificial urine solution was changed to alkaline conditions (pH 9.4) and the release of DF and IBU anti-inflammatory drugs was also investigated in such situation. In alkaline pH conditions (Figure 10a,b) it can be noticed that the release increased continuously until the end of the time period, although getting slower especially within the first 48 h. Besides, all the samples reached 100% of drug release at the end of the experiments. If the amount of drug released within 48 h is considered for each sample (see Table 3) it can be noticed that in alkaline conditions, most of the samples showed a limited burst release effect with respect to neutral pH. This aspect is noticed especially in the case of IBU release.

In order to explain the different release behaviors observed at different pH conditions, the chemical properties of both drug molecules should be considered. Both the DF and IBU drug used in this work are in the salt form and are characterized by an acid dissociation constant (pKa) equal to 4 at 25 °C. This quantity defines the capacity of a chemical specie to dissociate in ions upon interaction with a solution at a specific pH value. Therefore, from low pKa value it is possible to determine how strong is an acid. This dimension also depends on other physical quantities, as for instance the temperature, and in particular pKa increases with increasing of the latter. Hence, the pKa value of both drugs in salts form at 37 °C, i.e., the temperature of artificial urine solution used for release experiments, should be higher than 4. Usually, when the solution pH changes, as in this study, the balance of the dissolution reaction shifts, in accordance with the solute value of pKa. In particular, two conditions may occur, and they are reported below, where HA refers to the acid:

HA + H_2_O → A^−^ + H_3_O*^+^*

pH > pKa → [A^−^] > [HA] → dissociation of acid

pH < pKa → [HA] > [A^−^] → acid does not dissociate.

Comparing the maximum percentages of drug released in acid and alkaline pH conditions with respect to the physiological pH at 7, it can be noticed that these values are higher in case of alkaline pH and lower for acid pH. These differences occurred because in the first two studies (at pH 7 and 9.4), the solution had a pH >> pKa, which means that the drug was able to dissociate completely and to easily get free in the solution. By contrast, when the urine pH was 5, at 37 °C happened the case pH < pKa, which means that the drug dissolution was delayed and/or partially prevented.

### 3.5. Characterization of Post-Release Samples

**Figure 11:**
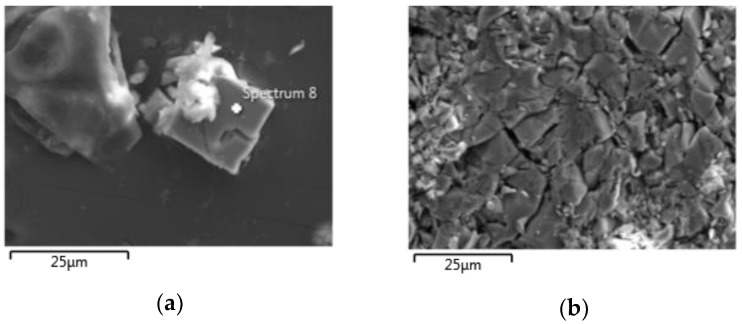
FESEM images of salts deposits and encrustations formed at the surface of the samples after 2 weeks of immersion in artificial urine: (**a**) polyHEMA@ZnO_0.1% and (**b**) polyHEMA@ZnO_1%.

**Figure 12:**
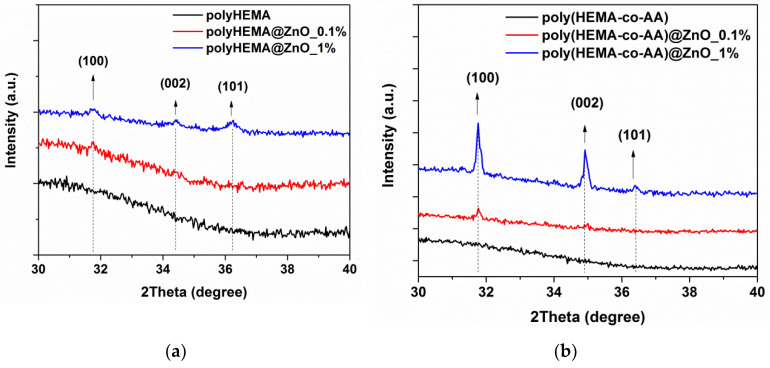
XRD pattern of (**a**) polyHEMA@ZnO and (**b**) poly(HEMA-co-AA)@ZnO samples at the end of the release study. Arrows indicate the diffraction peaks belonging to wurtzite ZnO phase.

After the release studies, FESEM and XRD analyses were carried out to check if some encrustations and deposition of salts occurred at the end of the immersion in AU. Besides, these analyses were taken also as a preliminary study on the degradation behavior of the investigated composite samples. Figure 11 shows the FESEM images of polyHEMA@ZnO_0.1% (panel a) and polyHEMA@ZnO_1% (panel b) acquired at the end of the studied period in AU at physiological pH conditions. The formation of encrustations and deposition of salts, mainly constituted by sodium and phosphate salts, after 14 days could be noticed from Table 4 in relatively low amount, alongside with the presence of Zn traces in the composition of the sample. These results witness that the degradation of the incorporated ZnO micropowders was not completed within two weeks of immersion in AU at physiological pH. This last aspect was further confirmed by XRD measurements (Figure 12), which indicated the presence of wurtzite ZnO powders within the composites even after the release study.

**Figure 13:**
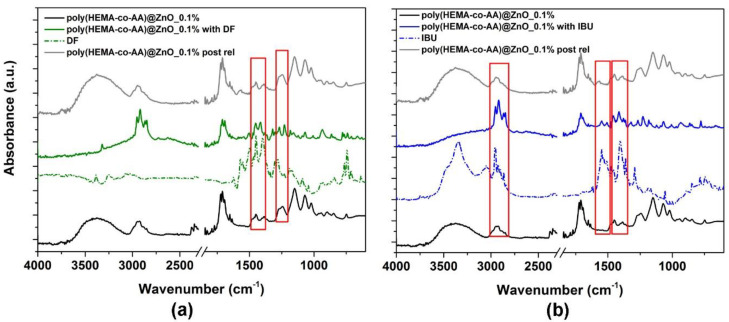
FTIR spectra of poly(HEMA-co-AA)@ZnO_0.1% samples in case of (**a**) Diclofenac release and (**b**) Ibuprofen release.

The FTIR analysis of the samples after two weeks of delivery in AU solution at physiological pH showed the complete release of both drugs. Figure 13 reports the representative FTIR spectra of the copolymer composites containing 0.1% of ZnO, i.e., sample polyHEMA@ZnO_0.1%, while the FTIR spectra for the other samples are shown in Figure S7 of the Supplementary Materials. From the starting spectrum of the composite (black curve), the effective loading of both IBU and DF is confirmed by the presence of typical IR vibration modes of each drug (panel a: green curve for DF uptake and dashed green curve for pure DF drug; panel b: blue curve for IBU uptake and dashed blue curve for pure IBU). After both drug release, the typical vibration of the two drugs are no longer visible (grey spectra), and the corresponding FTIR spectra appear very similar to the one of the starting composite materials, i.e., prior to the uptake of each drug.

Both for IBU and DF release it can also be observed, from the corresponding FTIR spectra (grey curves), that no particular signatures of deposited salts are visible. As this measurement is performed on a broad size of the sample surface, it collectively shows that the presence of salts and inorganic deposit previously observed, is limited to small portions of the sample surface and hence is not so pronounced, confirming the previous results. This further supports the idea that the proposed composite materials can show high potential in fabricating drug-eluting stent combining characteristics of biocompatibility, anti-bacterial properties and prevention of encrustation deposition at least for short time periods. Certainly, more detailed studies in this direction are foreseen.

## 4. Conclusions

New composite materials based on the incorporation of mesoporous flower-like ZnO micropowders into polyHEMA and poly(HEMA-co-AA) hydrogels were investigated for drug eluting stents applications. Crystalline ZnO powders with flower-like morphology, good surface area and mesopore size were prepared by a low-cost hydrothermal process. Then, composite materials were fabricated combining different percentages of the inorganic ZnO powder (0.1% and 1% w/v) during the polymerization process of both polyHEMA and poly(HEMA-co-AA). The morphology analysis and structural characterization pointed out the correct integration of ZnO material inside the composite while thermal analyses demonstrated the higher stability of the copolymer, which was even more improved thanks to the addition of ZnO.

Drug release experiments in artificial urine, even performed at acidic and alkaline pH conditions, evidenced that the amount of ZnO inside the formulation did not negatively affect the ability of the considered hydrogels to store and release the anti-inflammatory drugs. On the other hand, the incorporated ZnO micropowders may work as antimicrobial agents and are expected to confer the composite additional antibacterial properties with respect to the pure hydrogel. Moreover, depending on the type of hydrogel (polymer or copolymer), the stability and release kinetics of the composite in organic fluids changed during time. At physiological conditions, a sustained drug release was observed, with the pure hydrogels showing limited burst effects and kinetic constants as low as 0.05 h^−1^. When the pH conditions were changed from neutral to acidic/alkaline ones, the sustained release of both drugs was even more appreciable, with drug delivery starting after 4–24 h in some cases and a general reduction of the kinetic constant values, reaching the minimal values of 0.01–0.02 h^−1^ in the best cases. Furthermore, the release study pointed out that poly(HEMA-co-AA)@ZnO_0.1% is the material which has shown a good stability together with the best release trend in terms of low kinetic constant and limited burst release. Hence, this formulation satisfies the multifunctional requirements needed in the field of ureteral stent applications such as anti-bacterial effects, drug elution and biodegradability.

## Figures and Tables

**Table 1:** Kinetic parameters for DF and IBU drug release at physiological pH conditions.

pH	Composite Type		Diclofenac				Ibuprofen			
7.3	Polymer	ZnO, (w/v)	K, (h^−1^)	A_48h_, (%)	A, (%)	R^2^	K, (h^−1^)	A_48h_, (%)	A, (%)	R^2^
	polyHEMA	-	0.110	93	94	0.993	0.050	83	100	0.993
		0.1%	0.226	96	87	0.996	0.051	84	100	0.980
		1%	0.239	92	94	0.945	0.122	98	100	0.955
	polyHEMA-co-AA	-	0.178	93	94	0.997	0.050	87	100	0.992
		0.1%	0.180	100	100	0.998	0.070	91	100	0.919
		1%	0.212	100	100	0.998	0.121	96	100	0.998

**Table 2:** Kinetic parameters for DF and IBU drug release at acidic pH conditions.

pH	Composite Type		Diclofenac				Ibuprofen			
5.2	Polymer	ZnO, (w/v)	K, (h^−1^)	A_48h_, (%)	A, (%)	R^2^	K, (h^−1^)	A_48h_, (%)	A, (%)	R^2^
	polyHEMA	-	0.085	95	97	0.960	0.04	80	92	0.987
		0.1%	0.070	94	94	0.953	0.055	87	95	0.993
		1%	0.100	92	95	0.945	0.130	93	94	0.955
	polyHEMA-co-AA	-	0.06	75	60	0.975	0.012	97	90	0.999
		0.1%	0.08	100	74	0.990	0.060	94	100	0.957
		1%	0.05	100	92	0.975	0.080	99	100	0.998

**Table 3:** Kinetic parameters for DF and IBU drug release at alkaline pH conditions.

pH	Composite Type		Diclofenac				Ibuprofen			
9.4	Polymer	ZnO, (w/v)	K, (h^−1^)	A_48h_, (%)	A, (%)	R^2^	K, (h^−1^)	A_48h_, (%)	A, (%)	R^2^
	polyHEMA	-	0.12	94	100	0.927	0.025	68	100	0.972
		0.1%	0.15	93	100	0.950	0.036	76	100	0.939
		1%	0.100	91	100	0.945	0.025	70	100	0.873
	polyHEMA-co-AA	-	0.15	95	100	0.978	0.02	62	100	0.899
		0.1%	0.08	88	100	0.992	0.023	63	100	0.987
		1%	0.085	88	100	0.971	0.05	68	100	0.9748

**Table 4:** Energy-dispersive X-ray (EDX) results of the samples polyHEMA@ZnO_0.1% and polyHEMA@ZnO_1% after 2 weeks of immersion in artificial urine.

Element	polyHEMA@ZnO_0.1%	polyHEMA@ZnO_1%
	At.%	At.%
C	16.46	14.50
O	56.09	56.86
Na	8.76	12.09
Si	0.20	0.18
P	7.80	7.52
Zn	10.68	8.85
Total:	100.00	100.00

## Supplementary Materials

The following are available online at https://www.mdpi.com/1996-1944/13/17/3821/s1, Figure S1: Picture of sample polyHEMA@ZnO_1%; Figure S2: (a) Nitrogen sorption isotherm with indication of the calculated BET surface area and (b) DFT pore size distribution of the mesoporous ZnO flower-like microparticles; Figure S3: Morphological analysis of poly(HEMA-co-AA)@ZnO composite samples incorporating different ZnO amounts: (a) ZnO_0.1 wt.%; (b) ZnO_1 wt.%; Figure S4: EDX results obtained for (a) polyHEMA@ZnO_0.1% and (b) polyHEMA@ZnO_1%; Figure S5: FTIR spectrum of mesoporous ZnO flower-like powders; Figure S6: Concentration of zinc cations released from ZnO-based samples in cell culture medium; Figure S7: FTIR spectra in case of Diclofenac and Ibuprofen release: (a,b) polyHEMA; (c,d) polyHEMA@ZnO_0.1%; (e,f) polyHEMA@ZnO_1%; (g,h) poly(HEMA-co-AA); (i,j) poly(HEMA-co-AA)@ZnO_1%.
