# Supplementary material for: Biodegradable and Drug-Eluting Inorganic Composites Based on Mesoporous Zinc Oxide for Urinary Stent Applications
Source: Materials (Basel). 2020 Aug 29;13(17):3821. doi: 10.3390/ma13173821 (PMC7504493; doi:10.3390/ma13173821)
Supplement: Supplementary file 1 [file materials-13-03821-s001.pdf]

Supporting information

Biodegradable and drug-eluting inorganic composites based on mesoporous zinc oxide for urinary stent applications

Marco Laurenti, Marta Grochowicz, Elena Dragoni, Marco Carofiglio, Tania Limongi, and Valentina Cauda

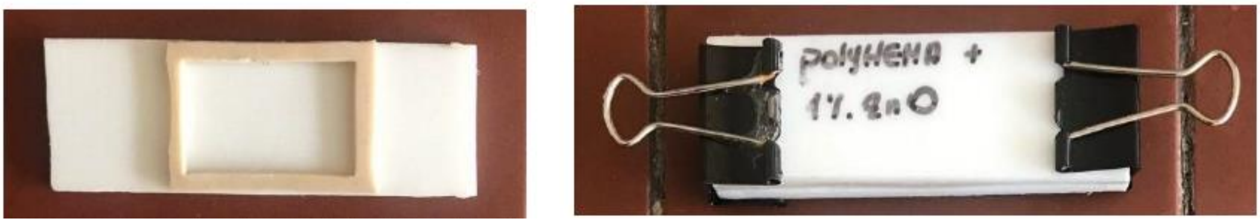

Figure S1. Picture of sample polyHEMA@ZnO_1% which includes the silicon rubber mold and plates of Teflon.

The nitrogen sorption measurement allows for the calculation with the Brunauer-Emmet-Teller (BET) model of the specific surface area, about 19.58 m^2/g. It also resulted some mesopourous-sized porosities of about 4 nm in diameter, calculated by Density Functional Theory (DFT) model applied to the equilibrium desorption branch of the isotherm. These pores, as well as the flower-like morphology, are responsible for the relatively high surface area and also act as preferential adsorption sites of the drugs.

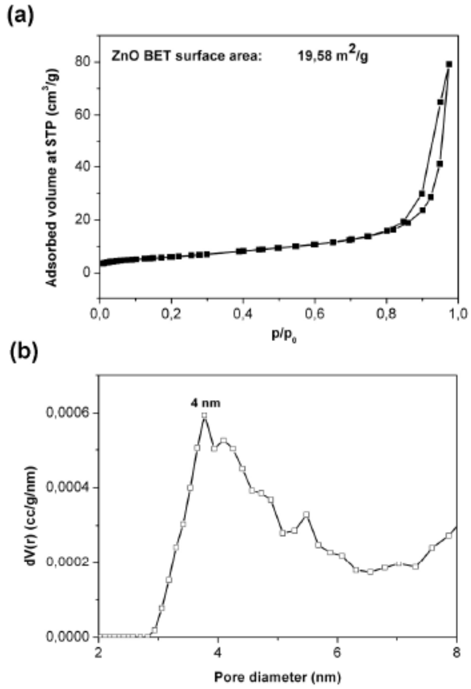

Figure S2. (**a**) Nitrogen sorption isotherm with indication of the calculated BET surface area and (**b**) DFT pore size distribution of the mesoporous ZnO flower-like microparticles.

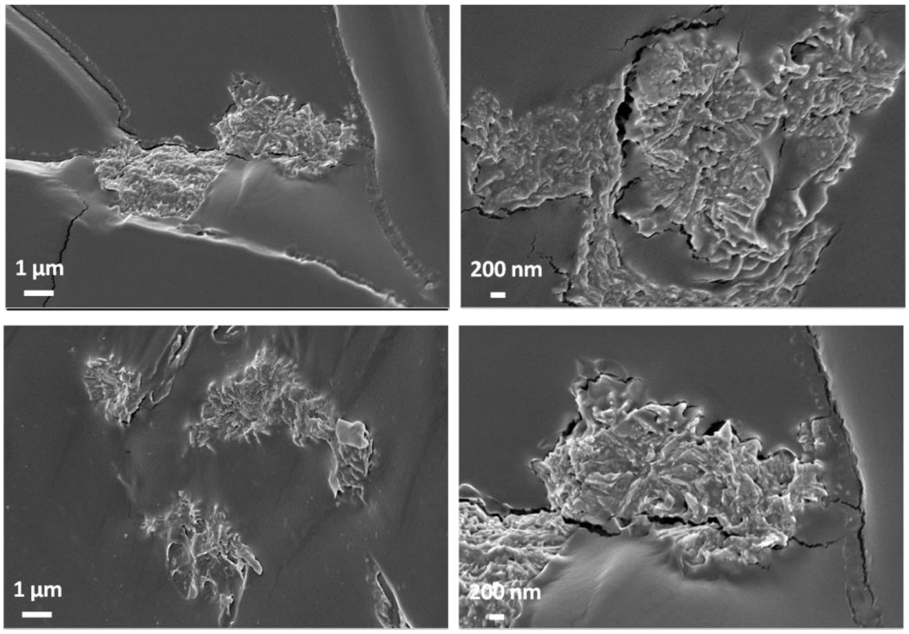

Figure S3. Morphological analysis of poly(HEMA-co-AA)@ZnO composite samples incorporating different ZnO amounts: (a) ZnO_0.1 wt%; (b) ZnO_1 wt%.

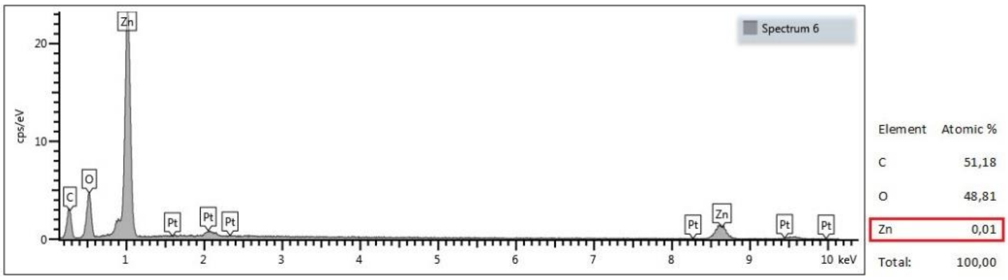

(a)

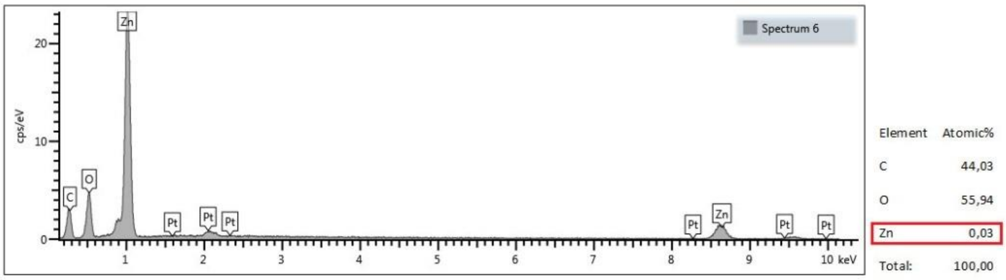

(b)

Figure S4. EDX results obtained for (**a**) polyHEMA@ZnO_0.1% and (**b**) polyHEMA@ZnO_1%. Each table summarizes the % atomic weight of each detected element. The detection of Pt is due to metallic coating of the samples needed for FESEM imaging.

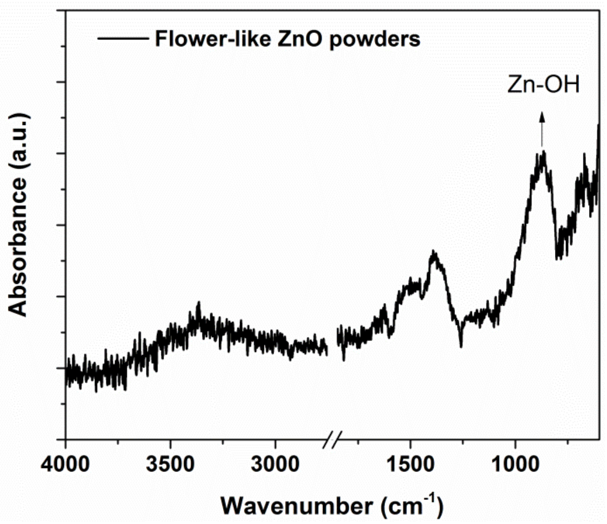

Figure S5. FT-IR spectrum of mesoporous ZnO flower-like powders.

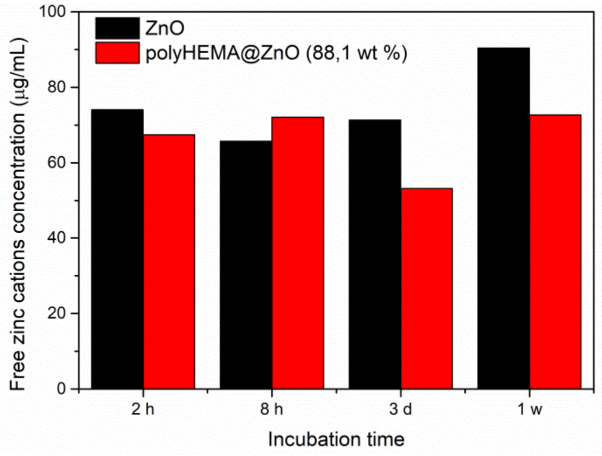

Figure S6. Concentration of zinc cations released from ZnO-based samples in cell culture medium (DMEM ,10 % fetal bovine serum) at different incubation times.

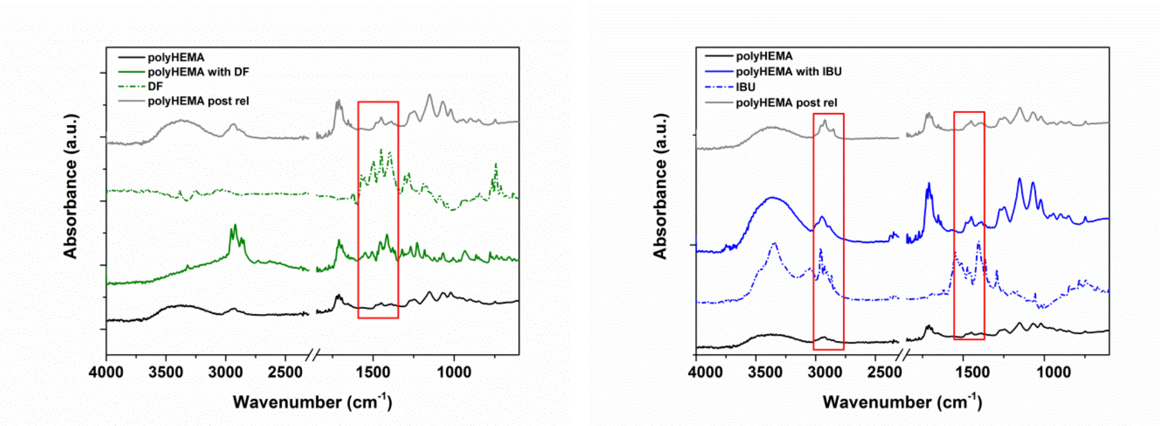

(**a**) (**b**)

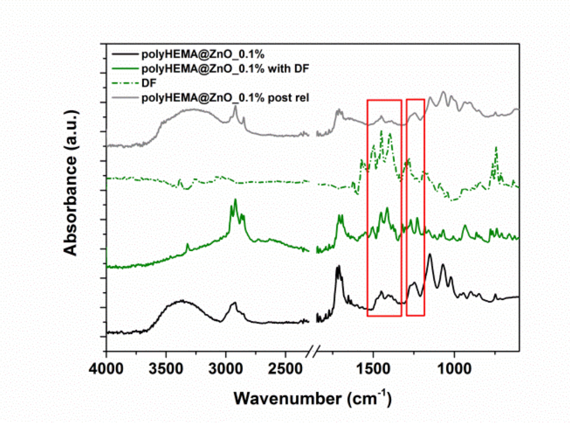
polyHEMA@ZnO_0.1%
polyHEMA@ZnO_0.1% with DF
DF
polyHEMA@ZnO_0.1% post rel
Absorbance (a.u.)
4000 3500 3000 2500 1500 1000
Wavenumber (cm-1)

(**c**)

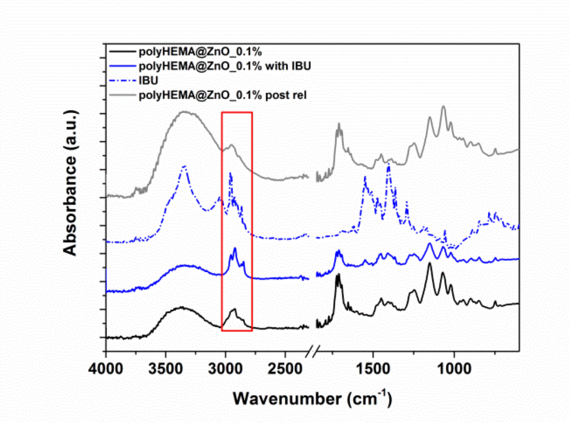
polyHEMA@ZnO_0.1%
polyHEMA@ZnO_0.1% with IBU
IBU
polyHEMA@ZnO_0.1% post rel
Absorbance (a.u.)
4000 3500 3000 2500 1500 1000
Wavenumber (cm-1)

(**d**)

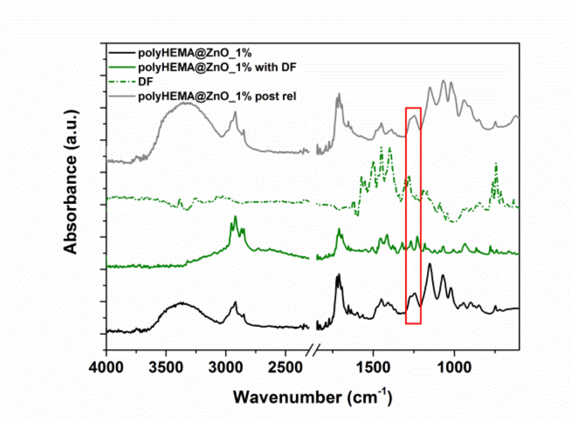
polyHEMA@ZnO_1%
polyHEMA@ZnO_1% with DF
DF
polyHEMA@ZnO_1% post rel
Absorbance (a.u.)
4000 3500 3000 2500 1500 1000
Wavenumber (cm-1)

(**e**)

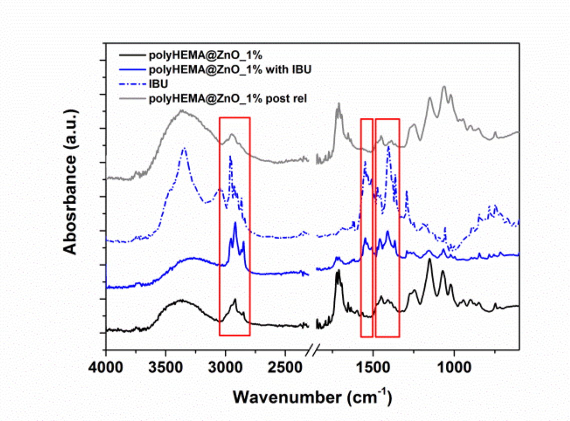
polyHEMA@ZnO_1%
polyHEMA@ZnO_1% with IBU
IBU
polyHEMA@ZnO_1% post rel
Absorbance (a.u.)
4000 3500 3000 2500 1500 1000
Wavenumber (cm-1)

(**f**)

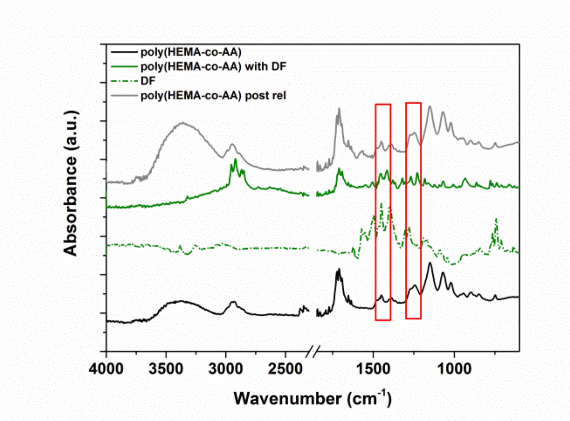
poly(HEMA-co-AA)
poly(HEMA-co-AA) with DF
DF
poly(HEMA-co-AA) post rel
Absorbance (a.u.)
4000 3500 3000 2500 1500 1000
Wavenumber (cm-1)

(**g**)

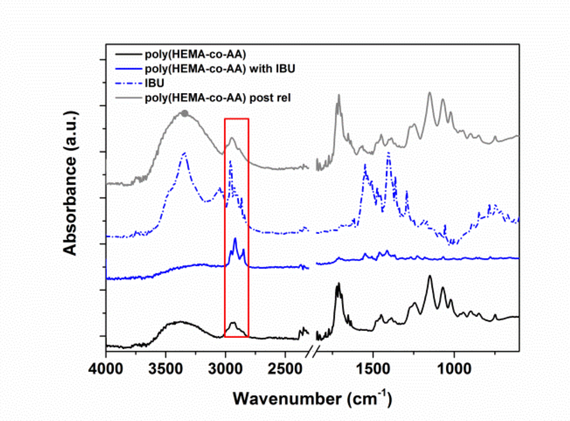
poly(HEMA-co-AA)
poly(HEMA-co-AA) with IBU
IBU
poly(HEMA-co-AA) post rel
Absorbance (a.u.)
4000 3500 3000 2500 1500 1000
Wavenumber (cm-1)

(**h**)

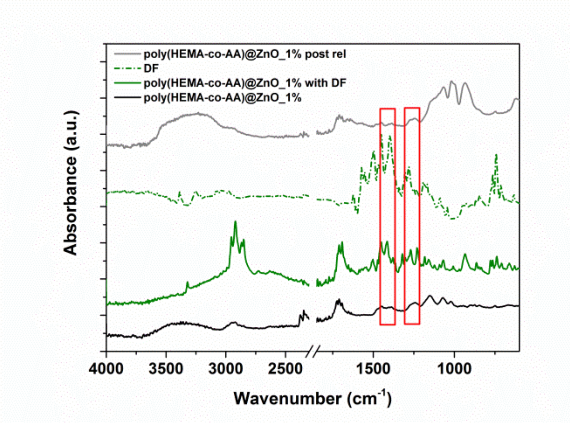

(**i**)

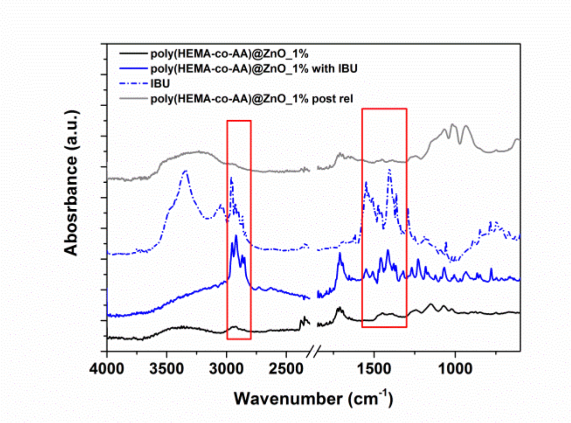

(**j**)

Figure S7. FT-IR spectra in case of Diclofenac and Ibuprofen release: (**a**,**b**) polyHEMA; (**c**,**d**) polyHEMA@ZnO_0.1%; (**e**,**f**) polyHEMA@ZnO_1%; (**g**,**h**) poly(HEMA-co-AA); (**i**,**j**)poly(HEMA-co-AA)@ZnO_1%.
